# Atractylenolide III ameliorates spinal cord injury in rats by modulating microglial/macrophage polarization

**DOI:** 10.1111/cns.13839

**Published:** 2022-04-10

**Authors:** Meng‐Tong Xue, Wen‐Jie Sheng, Xue Song, Yu‐Jiao Shi, Zhi‐Jun Geng, Lin Shen, Rui Wang, He‐Zuo Lü, Jian‐Guo Hu

**Affiliations:** ^1^ Department of Clinical Laboratory The First Affiliated Hospital of Bengbu Medical College Bengbu P.R. China; ^2^ 74539 Anhui Key Laboratory of Tissue Transplantation Bengbu Medical College Bengbu P.R. China; ^3^ 74539 Department of Immunology Bengbu Medical College Bengbu P.R. China

**Keywords:** atractylenolide III, microglia, neuroinflammation, spinal cord injury

## Abstract

**Background:**

Inflammatory reactions induced by spinal cord injury (SCI) are essential for recovery after SCI. Atractylenolide III (ATL‐III) is a natural monomeric herbal bioactive compound that is mainly derived in Atractylodes macrocephala Koidz and has anti‐inflammatory and neuroprotective effects.

**Objective:**

Here, we speculated that ATL‐III may ameliorate SCI by modulating microglial/macrophage polarization. In the present research, we focused on investigating the role of ATL‐III on SCI in rats and explored the potential mechanism.

**Methods:**

The protective and anti‐inflammatory effects of ATL‐III on neuronal cells were examined in a rat SCI model and lipopolysaccharide (LPS)‐stimulated BV2 microglial line. The spinal cord lesion area, myelin integrity, and surviving neurons were assessed by specific staining. Locomotor function was evaluated by the Basso, Beattie, and Bresnahan (BBB) scale, grid walk test, and footprint test. The activation and polarization of microglia/macrophages were assessed by immunohistofluorescence and flow cytometry. The expression of corresponding inflammatory factors from M1/M2 and the activation of relevant signaling pathways were assessed by Western blotting.

**Results:**

ATL‐III effectively improved histological and functional recovery in SCI rats. Furthermore, ATL‐III promoted the transformation of M1 into M2 and attenuated the activation of microglia/macrophages, further suppressing the expression of corresponding inflammatory mediators. This effect may be partly mediated by inhibition of neuroinflammation through the NF‐κB, JNK MAPK, p38 MAPK, and Akt pathways.

**Conclusion:**

This study reveals a novel effect of ATL‐III in the regulation of microglial/macrophage polarization and provides initial evidence that ATL‐III has potential therapeutic benefits in SCI rats.

## Graphical abstract

ATL‐III has potential therapeutic benefits in promoting histological and functional repair in rats after SCI. This effect may be partly mediated by inhibiting neuroinflammation induced by microglial polarization through the NF‐κB, JNK MAPK, p38 MAPK, and Akt pathways.

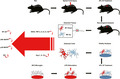

## 1 INTRODUCTION

Spinal cord injury (SCI) is caused by various factors and leads to sensory and motor dysfunction and eventually varying degrees of paralysis.^1^ The process of SCI includes two stages: primary injury induced by the initial impact and secondary injury resulting from inflammatory environmental factors.^2^ Although primary injury cannot be avoided, the biochemical and cellular events in the secondary injury stage are essential for recovery after SCI.^3^ Thus, the current clinical treatment strategies for SCI mainly involve ameliorating secondary injury, and identifying drugs that can effectively alleviate secondary injury is important.

In recent years, a large number of reports have confirmed that Chinese herbal medicines, especially natural monomers derived from plant extracts, effectively treat inflammation and immune‐related diseases with lower toxicity and drug resistance.^4^ Atractylenolide III (ATL‐III) is the main bioactive constituent of Atractylodes macrocephala Koidz and has a wide range of sources, such as Codonopsis pilosula and Chloranthus henryi Hemsl.^5^ According to reports, ATL‐III exhibits a series of pharmacological activities, including the inhibition of inflammation, antitumor and antiallergic responses, antagonizing apoptosis and neuroprotection.^5^, ^6^, ^7^ Wang et al^8^ found that ATL‐III could reduce mitochondrial damage and increase the activity of antioxidases to alleviate skeletal muscle atrophy induced by chronic kidney disease. Ji et al^5^ reported that ATL‐III can suppress nuclear factor‐κB (NF‐κB) p65 and mitogen‐activated protein kinase (MAPK) p38 signaling in mouse macrophages induced by lipopolysaccharide (LPS), exerting an anti‐inflammatory effect. Zhao et al^9^ showed that ATL‐III has an obvious neuroprotective effect against neuronal apoptosis induced by glutamate and may have therapeutic potential for nervous system diseases mediated by excitotoxicity. Furthermore, it was also reported that ATL‐III can significantly alleviate learning and memory dysfunction caused by long‐term administration of high‐dose homocysteine in animals and prevent apoptosis of primary cultured neurons induced by homocysteine.^7^ Recent studies have found that ATL‐III can alleviate cerebral ischemic injury by reducing neuroinflammation both in vivo and in vitro.^10^

Nevertheless, the role of ATL‐III in SCI remains unknown. This study examined the effect of ATL‐III in ameliorating SCI and explored the underlying molecular mechanisms *in vivo* and *in vitro*, providing new insights into SCI treatment.

## 2 MATERIALS AND METHODS

### 2.1 Animals

Sprague–Dawley (SD) rats (female rat, 8 weeks old, 220–250 g) were obtained from Changzhou Cavens Laboratory Animals Ltd. All animal husbandry procedures were executed according to the Administration of Affairs Concerning Experimental Animals Guidelines (Ministry of Science and Technology; 2004), and all experiments were performed according to ARRIVE guidelines.^11^

### 2.2 Contusive SCI and drug administration

Seventy‐two rats were randomly divided into 3 groups: a sham group (Sham), a control group (Ctrl), and an ATL‐III treatment group (ATL‐III). Contusion SCI was established with an Infinite Horizon impactor (Precision Systems).^12^ In brief, the anaesthetization of rat was executed with pentobarbital (50 mg/kg, i.p.), and a T9 vertebral laminectomy was performed. Moderate SCI was established with a 2.5‐mm‐diameter rod (force 120 kdynes). The sham rats underwent the same surgical procedure without contusion injury. Postoperatively, the rats were placed in a room with controlled conditions (20 ± 2°C, 55 ± 10% humidity). To protect against infection, the rats were administered 50 mg/kg chloramphenicol (Sangon Biotech) by drinking water. ATL‐III (Phytomarker Ltd.) was mixed with 0.9% NaCl and dimethyl sulfoxide (DMSO, 1%). The rats were administered 5 mg/kg ATL‐III by gavage three hours after the operation and then once a day until sacrifice.^6^, ^7^, ^8^ The control rats were administered the same volume of saline.

### 2.3 Behavioral assessments

Animals that survived for more than 42 days underwent the grid walking test, the open field test, and footprint analysis as previously described.^13^, ^14^, ^15^ The behavioral tests were performed by 2 trained investigators who were blinded to the animal grouping information.

An open field and the Basso, Beattie, and Bresnahan (BBB) locomotor scale were used to evaluate locomotor recovery at specific time points. The crawling ability of the rats was scored on a scale of 0 to 21 over 4 min.

The grid walking test was carried out using a 100 × 100 cm grid runway with 50 × 50 mm holes formed by the rails of the grid. The rats were allowed to walk freely on the grid runway for 4 min each trial. At least 2 min of continuous walking time was required for analysis. When the paws and heel of the hind limb fell completely below the grid, an error was scored. Two investigators counted the total number of footfalls for one hind limb and the number of errors and calculated the mean error rate.

Footprint analysis was performed by evaluating the forepaw and hindpaw prints made by rats with red and blue ink as they passed through a narrow runway. To assess the weight support and gait of the rats, a 4‐point scoring system was used as described previously: 0, persistent limping or hind leg dragging, inability to make visible footprints; 1, visible toe prints for at least 3 toes in at least 3 footprints; 2, footprint showing exo‐ or endo‐rotation more than double the baseline angle; 3, no obvious dragging, only exo‐ or endo‐rotation; 4, no rotation.

### 2.4 Tissue preparation

At predetermined time points after SCI, the rats were anesthetized. The hearts of the rats were perfused with phosphate‐buffered saline (PBS) and then with 4% paraformaldehyde (PFA). After perfusion, a 10‐mm segment of the spinal cord that included the injury epicenter was removed (or the same spinal segments for sham rats), postfixed in 4% PFA, and then frozen in OCT (TissueTek, Miles, Elkart, IN).

### 2.5 Histological analysis

At 42 days postinjury, tissue from the remaining rats was obtained and sectioned as described in the “Tissue preparation” section. Luxol fast blue (LFB), hematoxylin–eosin (HE) and Nissl staining (Beyotime Biotechnology, China) were performed according to the manufacturer's instructions to assess the damaged area, the medullary white matter area and the number of remaining motor neurons as previously described.^16^ Nine cross‐sectional images were collected from the lesion epicenter (the section containing the largest lesion area and the least sparing of white matter), and light microscopy was used to visualize the sections 1, 2, 3, and 4 mm rostral and caudal to the epicenter. The lesion area and medullary white matter area were measured using ImageJ software (National Institutes of Health) and Image‐Pro 5.1 (Media Cybernetics), respectively, and normalized to the percentage of the entire stained area in an unbiased stereological manner for quantitative analysis.^16^ Surviving neurons were quantified by counting the number of Nissl bodies.

### 2.6 Cell culture and drug treatment

BV‐2 microglial cells (BV2, Cellcook) were cultured in DMEM supplemented with 10% FBS and 1% streptomycin/penicillin (Gibco) in a humidified atmosphere containing 5% CO_2_ at 37°C. ATL‐III (1, 10, or 100 µM) was mixed with culture medium containing 0.1% DMSO. According to our pilot experiment, cells were passaged in 6‐well plates (2 × 10^5^) or 96‐well plates (0. × 10^5^). After 24 h, ATL‐III was applied for 1 h, and then LPS (100 ng/ml, Sigma–Aldrich) was added to stimulate the cells for 24 h.^17^

### 2.7 Cell viability assay

The Cell Counting Kit‐8 (CCK‐8) assay (Vazyme Biotech) was used to evaluate the viability of BV2 cells in the ATL‐III (1, 10, or 100 µM) and vehicle groups. Briefly, when the BV2 cells reached 60% confluence, the culture medium was replaced with fresh medium containing different doses of ATL‐III (1, 10, or 100 µM), and the cells were incubated for 24 h. The cells were then cultured with fresh medium containing CCK‐8 reagent for 2 h. Finally, the absorbance was measured at 450 nm with a microplate reader (Thermo Fisher Scientific).

### 2.8 Immunohistofluorescence

At 7 days postinjury, the spinal cords of the rats were extracted to prepare frozen sections as described above. For immunohistochemical staining, the sections were thawed at 37°C, washed with PBS, and blocked with 1% serum (Sigma–Aldrich) at 37°C for 1 h. The sections were incubated with antibodies (Table 1) for 12 h at 4°C. The next day, the sections were washed with PBS and then incubated with secondary antibodies at 37°C for 1 h. Finally, a ZEISS Axio observation microscope with Zen imaging software (Carl Zeiss AG) was used to acquire and analyze images. The cells were counted as described in a previous report.^18^

**TABLE 1:** Table of antibodies used

Antigen	Host spices and clone	Cat. # or Lot #	RRID	Conjugation	Source	Concentration used
CD11b	Rabbit polyclonal	PA5‐79533	AB_2746648		Invitrogen	1:200
CD11b	Mouse monoclonal	12‐0110‐82	AB_11150971	PE	Invitrogen	0.5 μg/test
CD68	Mouse monoclonal	MA5‐13324	AB_10987212		Invitrogen	1:200
CD68	Mouse polyclonal	MCA341A647	AB_566874	Alexa Fluor 647	Bio‐rad	0.5 μg/test
CCR7	Rabbit monoclonal	MA5‐31992	AB_2809286		Invitrogen	1:200
CCR7	Rabbit polyclonal	bs−1305R	AB_11056896	PE	Bioss	1 μg/test
Arg−1	Rabbit polyclonal	PA5‐29645	AB_2547120		Invitrogen	1:200
CD206	Rabbit polyclonal	bs−4727R	AB_2894915	FITC	Bioss	1 μg/test
iNOS	Rabbit polyclonal	PA1‐036	AB_325773		Invitrogen	1:500
TNF‐α	Rabbit polyclonal	ab66579	AB_1310759		Abcam	1:1000
IL−1β	Rabbit polyclonal	ab9722	AB_308765		Abcam	1:1000
IL−6	Rabbit polyclonal	ab229381	AB_2861234		Abcam	1:1000
IL−10	Rabbit polyclonal	ab9969	AB_308826		Abcam	1:1000
p‐IκBα	Rabbit monoclonal	#2859	AB_561111		Cell Signaling	1:1000
IκBα	Rabbit monoclonal	#4812	AB_10694416		Cell Signaling	1:1000
p‐p65	Rabbit monoclonal	#3033	AB_331284		Cell Signaling	1:1000
p65	Rabbit monoclonal	#8242	AB_10859369		Cell Signaling	1:1000
p‐p38	Rabbit monoclonal	#4511	AB_2139682		Cell Signaling	1:1000
p−38	Rabbit monoclonal	#8690	AB_10999090		Cell Signaling	1:1000
p‐JNK	Rabbit monoclonal	#4668	AB_823588		Cell Signaling	1:1000
JNK	Rabbit monoclonal	#9252	AB_823588		Cell Signaling	1:1000
p‐ERK	Rabbit monoclonal	#4370	AB_2315112		Cell Signaling	1:2000
ERK	Rabbit monoclonal	#4695	AB_390779		Cell Signaling	1:1000
p‐AKT	Rabbit monoclonal	#4060	AB_2315049		Cell Signaling	1:2000
AKT	Rabbit monoclonal	#4691	AB_2225340		Cell Signaling	1:1000
β‐actin	Rabbit monoclonal	#8457	AB_10950489		Cell Signaling	1:1000
Mouse IgG (H + L)	Goat polyclonal	115‐095‐003	AB_2338589	Fluorescein (FITC)	Jackson ImmunoResearch	1:200
Mouse IgG (H + L)	Donkey polyclonal	715‐025‐151	AB_2340767	Rhodamine (TRITC)	Jackson ImmunoResearch	1:200
Rabbit IgG (H + L)	Goat polyclonal	111‐025‐144	AB_2337932	Rhodamine (TRITC)	Jackson ImmunoResearch	1:200
Rabbit IgG (H + L)	Donkey polyclonal	711‐097‐003	AB_2340598	Fluorescein (FITC)	Jackson ImmunoResearch	1:200
Rabbit IgG (H + L)	Goat polyclonal	BL003A	AB_2827666	HRP	Biosharp	1:2000

### 2.9 Flow cytometry

To obtain spinal cord tissue, rats were anesthetized 7 days following surgery and then perfused with PBS. A 10‐mm segment of the spinal cord, including the injury epicenter, was removed and dissociated into a single‐cell suspension by sufficient trituration as previously described.^19^ To analyze the proportion of microglial subtypes, the cell suspension was incubated with antibodies (Table 1) for 30 min at 4°C. Finally, a flow cytometer (Becton Dickinson) was used for detection, and the data were analyzed with FlowJo 7.6.1 software (FlowJo).

### 2.10 Western blot analysis

Ten‐millimeter spinal cord segments were extracted from rats euthanized as described above at 7 days postinjury. Protein was extracted from the spinal cord segments using radioimmunoprecipitation (RIPA) lysis buffer (Beyotime Biotechnology) containing phosphatase inhibitor cocktail (1:50; Beyotime Biotechnology) and protease inhibitors (1:100; Thermo Fisher Scientific) after they were fully homogenized. A bicinchoninic acid protein assay kit (Beyotime Biotechnology) was employed to quantify the protein concentration. The Western blot membranes were incubated with primary antibodies (Table 1) at 4°C overnight. After that, the membranes were washed with Tris‐buffered saline‐Tween‐20 (TBST, Biosharp) and incubated with secondary antibody at 37°C for 2 h. After incubation, the membranes were washed against with TBST three times at 37°C for 10 min, and a chemiluminescence kit (Thermo Fisher Scientific) was employed to visualize the immunoreactivity of the target proteins. The data were acquired and quantified using a ChemiDoc XRS imaging system (Bio–Rad) and ImageJ software (Bio–Rad). The gray values of specific bands were analyzed by normalizing their levels to β‐actin (Table 1).

### 2.11 Statistical analysis

AII results are presented as the mean ± SD. For two‐group comparisons, Student's *t* test or Mann–Whitney *U* (nonparametric tests) was used. One‐way anova or Kruskal–Wallis test (nonparametric tests) followed by the Bonferroni test was used for multiple groups. Nonparametric tests were conducted because of the nonnormal distribution and nonhomogeneity of variance. SPSS software v.14.0 (SPSS Inc.) was employed for statistical analysis, and the differences were defined as *p* < 0.05.

## 3 RESULTS

### 3.1 *ATL*‐*III ameliorated the motor deficits after SCI in rats*

**FIGURE 1:**
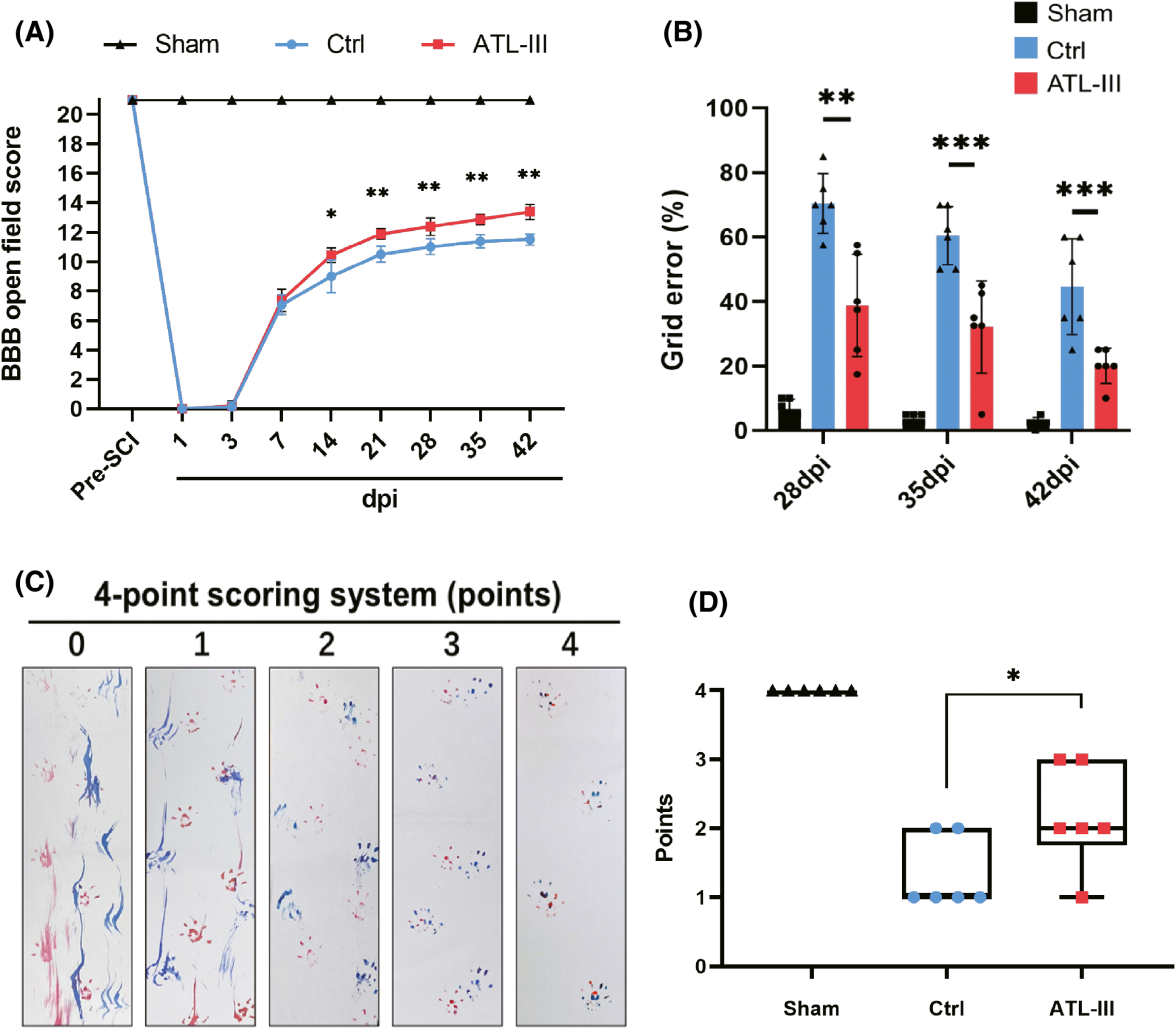
Effects of ATL‐III on the recovery of motor function in rats after SCI. (A) Comparative chart of BBB scores. (B) Performance in the grid walk test at 28, 35, and 42 days postinjury. (C,D) Footprint analysis at 42 days postinjury. The data are presented as the mean ± SD (*n* = 6). *p < 0.05, **p < 0.01, ***p < 0.001 compared with the control rats

Motor function was assessed at designated time points after SCI by the BBB locomotor scale, the grid walk test and footprint analysis. At 14–42 days following injury, the BBB scores of the ATL‐III group were obviously higher than those of the control rats (all *p* < 0.05; Figure 1A). The ability to control the hindlimbs was examined by the grid walk test at 28, 35 and 42 days postinjury. As shown in Figure 1(B), the error rate (rate of footfalls on the grid) in the ATL‐III group was observably lower than that in the control rats (all *p* < 0.01). Likewise, the ATL‐III group scored higher in the footprint analysis than the control group at day 42 (*p* < 0.05; Figure 1C,D). These data demonstrated that ATL‐III administration could observably improve motor function in an SCI model.

### 3.2 *ATL*‐*III attenuated spinal cord tissue damage*

**FIGURE 2:**
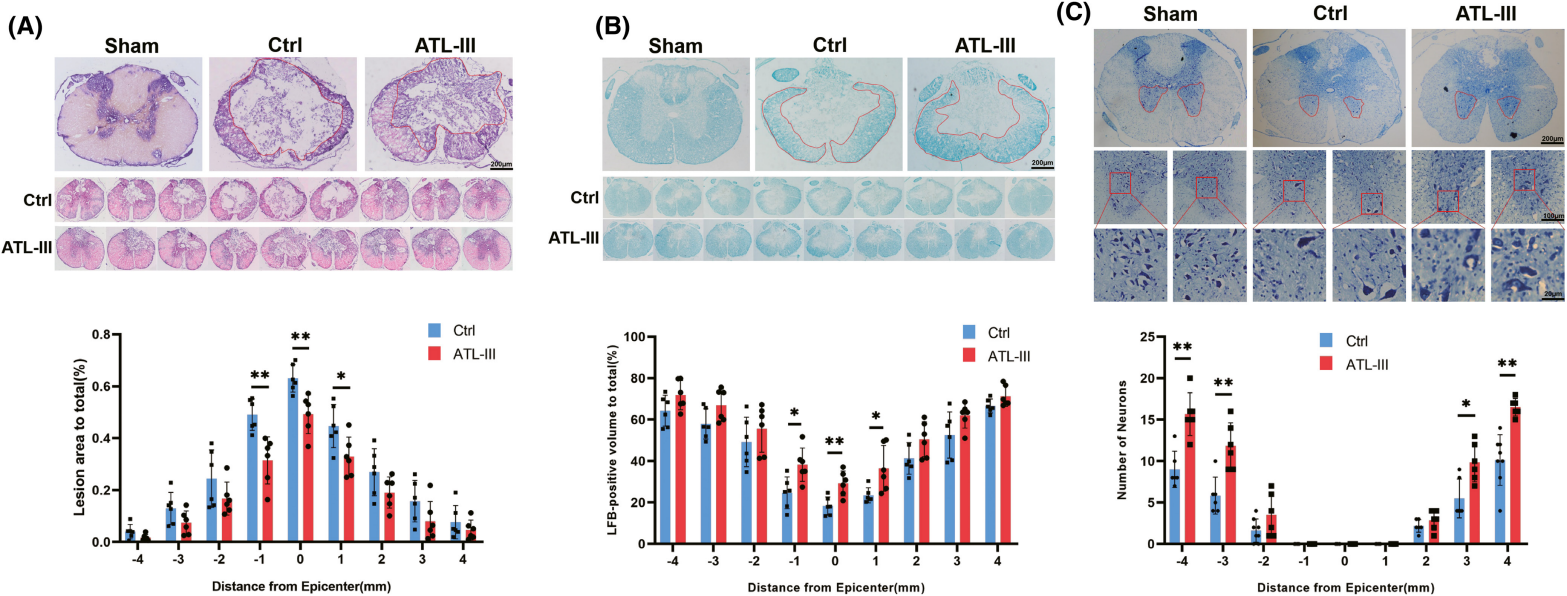
ATL‐III reduced the lesion area, increased residual myelination and alleviated motor neuron loss in rats after SCI. (A) Typical images of HE staining in the three groups and the lesion areas in the ATL‐III rats and the control rats. (B) Representative images of LFB staining in the three groups and quantitative analysis of residual myelination in the ATL‐III rats and the control rats. (C) Representative Nissl staining image of neurons and quantitative analysis of the number of motor neurons in the ATL‐III rats and the control rats. The data are presented as the mean ± SD (*n* = 6). *p < 0.05, **p < 0.01 compared to the control rats

To assess the effects of ATL‐III on histopathology following SCI, the lesion area, myelinated white matter, and remnant motor neurons were measured by HE, LFB, and Nissl staining. The images suggested that rats in the ATL‐III group had an observably smaller lesion area and larger LFB‐positive region at the epicenter (0) and the lateral (+) and caudal (−) sides 1 mm from the epicenter compared to those in the control rats (all *p* < 0.05; Figure 2A,B). The numbers of remaining motor neurons 3 and 4 mm rostral and caudal to the epicenter of the ATL‐III rats were greater than those in the control rats (all *p* < 0.05; Figure 2C).

### 3.3 *ATL*‐*III reduced the ratio of activated microglia*/*macrophages in SCI rats*

**FIGURE 3:**
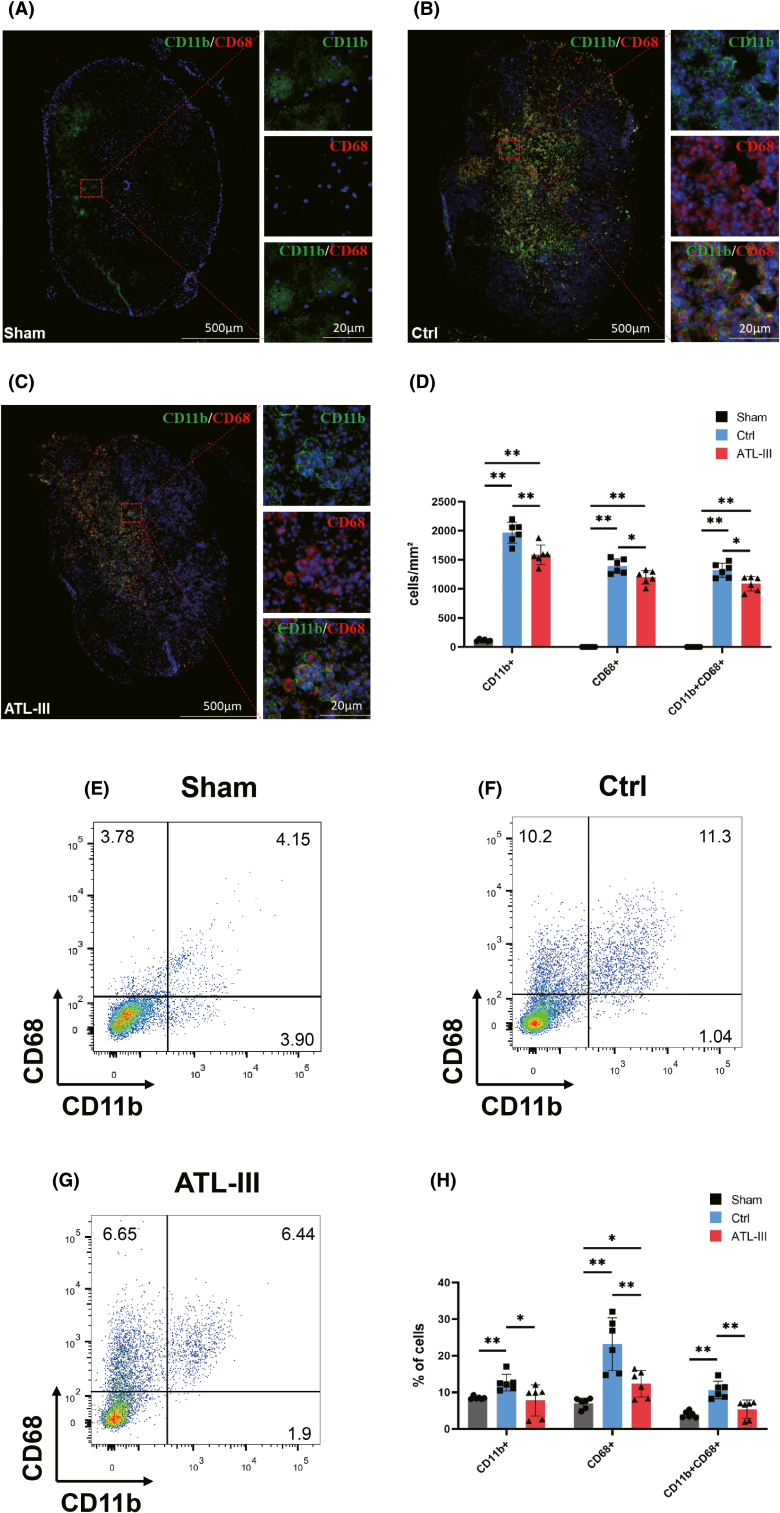
ATL‐III reduced the number of activated microglia/macrophages in SCI rats, as determined by immunohistofluorescence and flow cytometry. (A–C) Representative images of CD11b (green), CD68 (red) and nuclei (blue) in the spinal cords of sham, control, and ATL‐III rats. (D) The numbers of CD11b^+^, CD68^+^ and CD11b^+^CD68^+^ cells. (E–G) Typical flow cytometry images of myeloid tissues from sham, control, and ATL‐III rats. (H) Proportions of CD11b^+^, CD68^+^, and CD11b^+^CD68^+^ cells. The data are presented as the mean ± SD (*n* = 6). *p < 0.05, **p < 0.01

To identify the effect of ATL‐III on the status and number of microglia/macrophages in SCI rats, we performed immunohistofluorescence (IF) and flow cytometry (FCM). CD11b is a maker of microglia/macrophages, and CD68 is a maker of activated microglia/macrophages; thus, CD11b^+^CD68^+^ cells are activated microglia/macrophages.^19^, ^20^ As shown in Figure 3(A), CD68^+^ cells were very scarce in the sham rats, while there was an observably increase in the number of these cells in the control and ATL‐III rats (all *p* < 0.01; Figure 3D). The number of CD68^+^ cells was obviously lower in the ATL‐III rats than in the control rats (*p* < 0.05; Figure 3D). As shown in Figure 3(B,C), the number of CD11b^+^ cells was obviously increased in the control and ATL‐III rats compared with the sham rats (all *p* < 0.01; Figure 3D). In the sham group, typical resting microglia/macrophages had small somas and many tiny processes (Figure 3A). Following SCI, microglia/macrophage cells were activated and had larger and rounder somas (Figure 3B,C). Furthermore, the number of CD11b^+^ cells was decreased in the ATL‐III rats compared with the control rats (*p* < 0.01; Figure 3D). As shown in Figure 3A, CD11b^+^CD68^+^ cells were virtually absent in the sham rats, and the number of these cells was obviously increased in the control and ATL‐III rats (*p* < 0.01; Figure 3D). Likewise, the number of CD11b^+^CD68^+^ cells was lower in the ATL‐III rats than in the control rats (*p* < 0.05; Figure 3D).

The status and proportion of microglia/macrophages were further assessed by FCM. As shown in Figure 3(E–G), the ratios of all cells were lower in the sham rats than in the control rats (all *p* < 0.01; Figure 3H). Likewise, the ratios of all cells were obviously lower in the ATL‐III rats than in the control rats (all *p* < 0.05; Figure 3H). These results revealed that microglia/macrophages were activated and cell numbers were significantly increased following SCI and that ATL‐III decreased the number and proportion of activated microglia/macrophages.

### 3.4 *ATL*‐*III regulated M1*/*M2 differentiation of activated microglia*/*macrophages in SCI rats*

**FIGURE 4:**
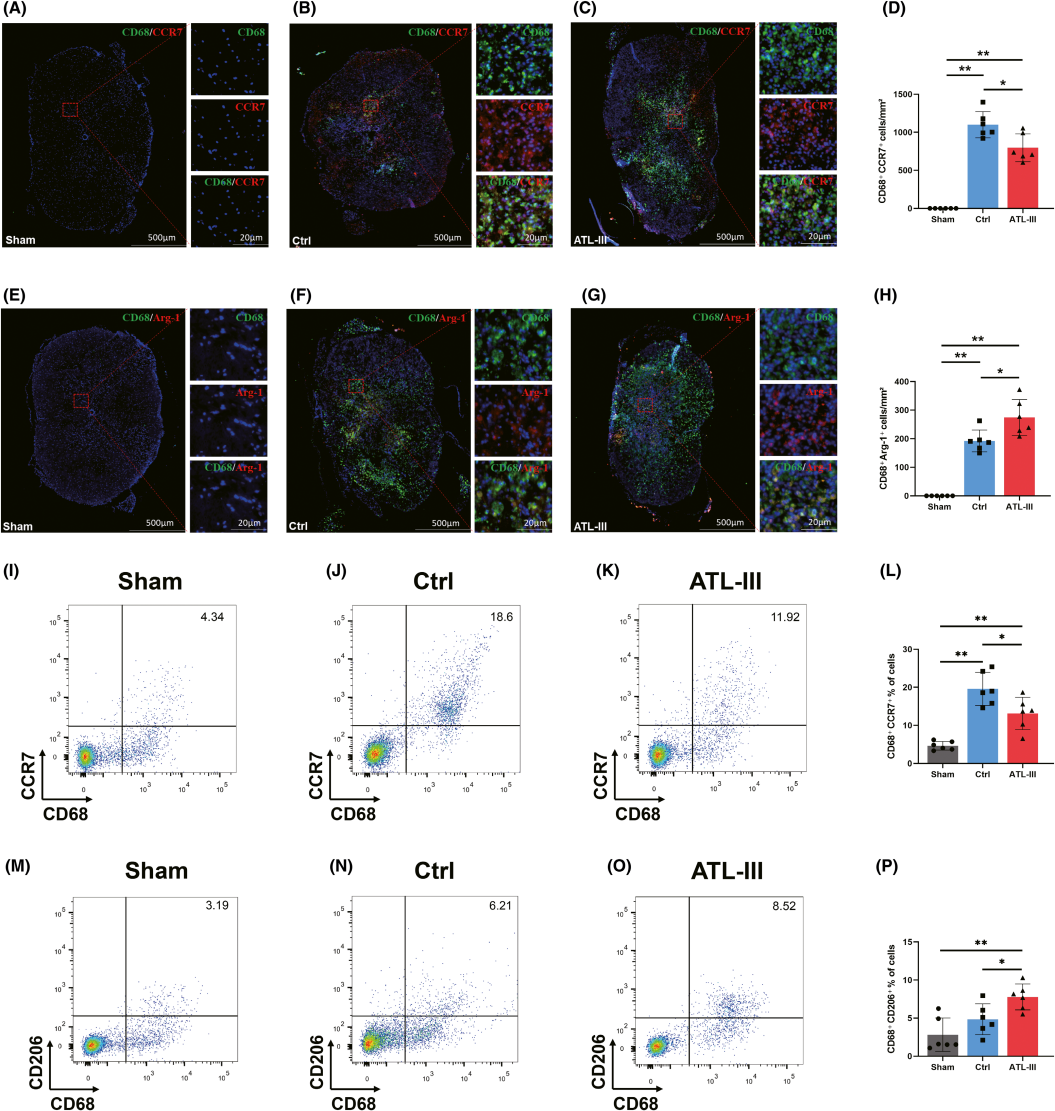
ATL‐III regulated the M1/M2 polarization of microglia/macrophages in rats after SCI. (A–H) Typical immunohistofluorescence pictures of CD68 (green), CCR7 (red in A–C), Arg‐1 (red in E–G), and nuclei (blue) in the spinal cords of sham, control, and ATL‐III rats. (D,H) The numbers of CD68^+^CCR7^+^ (M1 cells) and CD68^+^Arg‐1^+^ (M2 cells) cells. (I–K, M–O) Typical flow cytometry images of myeloid tissues from sham, control, and ATL‐III rats. (L,P) Proportions of CD68^+^CCR7^+^ and CD68^+^CD206^+^ cells. The data are presented as the mean ± SD (*n* = 6). *p < 0.05, **p < 0.01 compared with the control rats

To determine the effect of ATL‐III on M1/M2 differentiation in SCI rats, we labeled M1 and M2 cells with CD68, C‐C chemokine receptor type 7 (CCR7), arginase‐1 (Arg‐1) and CD206 and detected them via IF and FCM.^21^, ^22^ As shown in Figure 4(A–C), CD68^+^CCR7^+^ M1 cells were rare in the sham rats, but the number of these cells was obviously increased in the control and ATL‐III rats (*p* < 0.01; Figure 4D). There were fewer CD68^+^CCR7^+^ M1 cells in the ATL‐III rats than in the control rats (*p* < 0.05; Figure 4D). As shown in Figure 4(E–G), CD68^+^Arg1^+^ M2 cells were not detected in the sham rats, but the number of these cells was significantly increased in the control and ATL‐III groups (*p* < 0.01; Figure 4H). Remarkably, the number of CD68^+^Arg1^+^ M2 cells in the ATL‐III group was further significantly increased compared with that in the control rats (*p* < 0.05; Figure 4H).

To determine the effect of ATL‐III on the proportion of M1/M2 differentiation in SCI rats, the panel of CD68, CCR7, and CD206 molecules was further evaluated by FCM. CD68^+^CCR7^+^ cells were defined as M1 phenotypes, and CD68^+^CD206^+^ cells were defined as M2 phenotypes. As shown in Figure 4(I–K), the proportion of M1 cells was observably increased in the SCI rats compared with the sham rats (*p* < 0.01; Figure 4L). The proportion of M1 cells was lower in the ATL‐III rats than in the control rats (*p* < 0.05; Figure 4L). In contrast, the proportion of M2 cells was significantly higher in the ATL‐III rats than in the control rats (*p* < 0.01; Figure 4P).

These results suggested that the proportions of M1 and M2 cells are balanced in the normal spinal cord and that most microglia/macrophages are polarized toward the M1 phenotype postinjury. ATL‐III could regulate the M1/M2 differentiation of microglia by suppressing their polarization toward the M1 phenotype and enhancing their polarization toward the M2 phenotype.

### 3.5 *ATL*‐*III regulates the expression of corresponding inflammatory factors of M1*/*M2*

**FIGURE 5:**
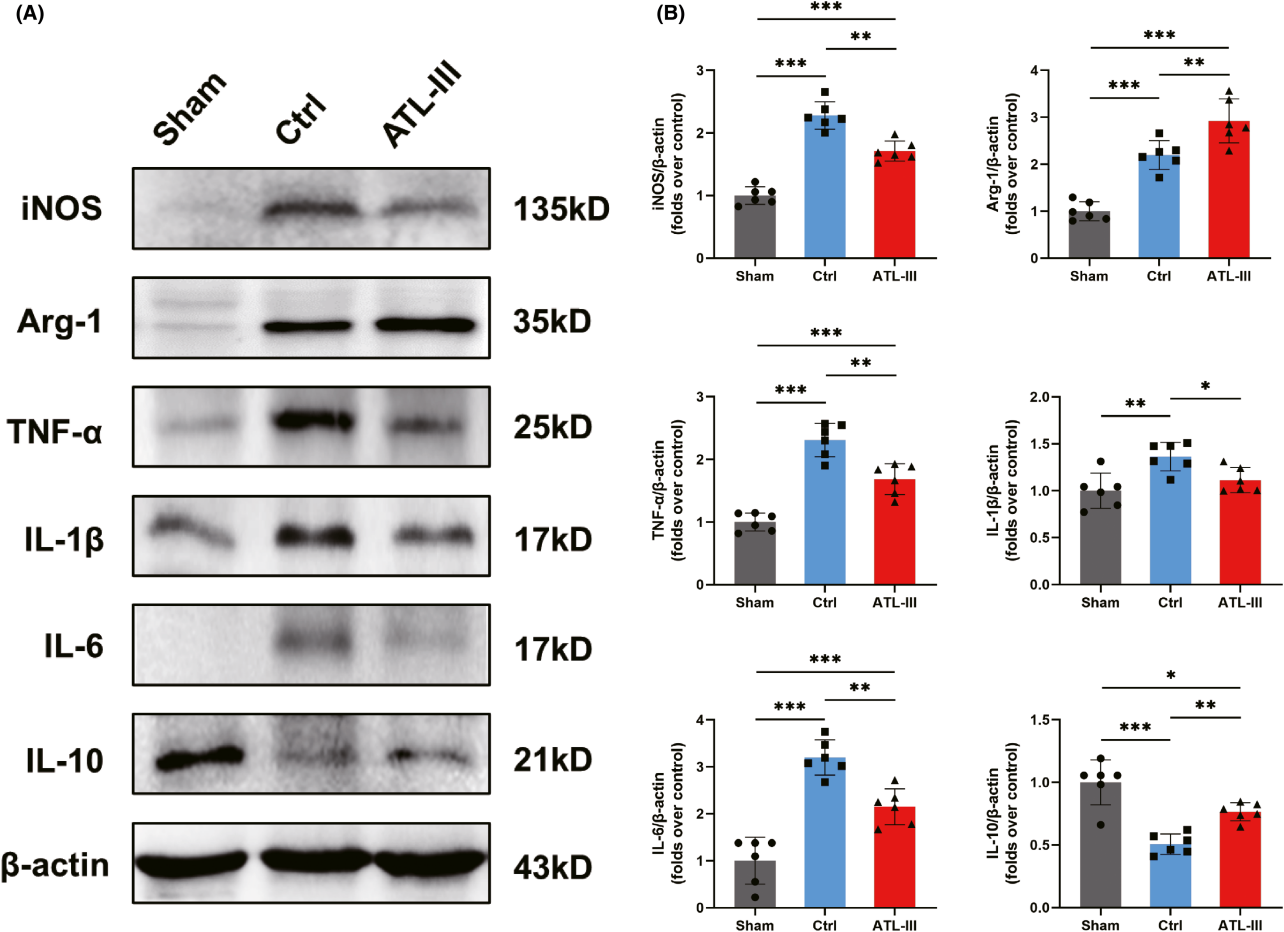
ATL‐III regulates the expression of the corresponding inflammatory factors from M1/M2 macrophages. (A) Western blot analysis iNOS, Arg‐1, inflammatory mediator (TNF‐α, IL‐1β and IL‐6), IL‐10 and β‐actin expression. (B) Quantitative analysis of protein expression levels. The data are presented as the mean ± SD (*n* = 6). *p < 0.05, **p < 0.01, ***p < 0.001 compared with the control rats

To further elucidate the protective mechanism of ATL‐III in SCI, the numbers of the M1/M2 cells and the level of corresponding inflammatory factors were analyzed by Western blotting. As shown in Figure 5(A,B), the level of inducible nitric oxide synthase (iNOS), which is a maker of the M1 phenotype,^23^ was observably increased in control rats (*p* < 0.001) but was decreased in ATL‐III rats (*p* < 0.01). The level of Arg‐1 was increased in both control rats and ATL‐III rats compared with sham rats (*p* < 0.001). The production of proinflammatory mediators, including tumor necrosis factor‐α (TNF‐α), interleukin (IL)‐1β, and IL‐6, was increased significantly in control rats compared with sham rats (all *p* < 0.01), and the production of these proinflammatory factors significantly decreased after ATL‐III administration (all *p* < 0.05). However, the production of the anti‐inflammatory factor IL‐10 by M2 macrophages was significantly decreased after SCI (*p* < 0.001) and increased significantly after ATL‐III administration, as expected (*p* < 0.01). These data suggest that the protective functions of ATL‐III in SCI rats and the expression of inflammatory factors are related to the regulation of the microglia/macrophage‐activated phenotype.

### 3.6 *ATL*‐*III regulates the M1*/*M2 phenotype and corresponding inflammatory factor expression after LPS stimulation in microglia*

**FIGURE 6:**
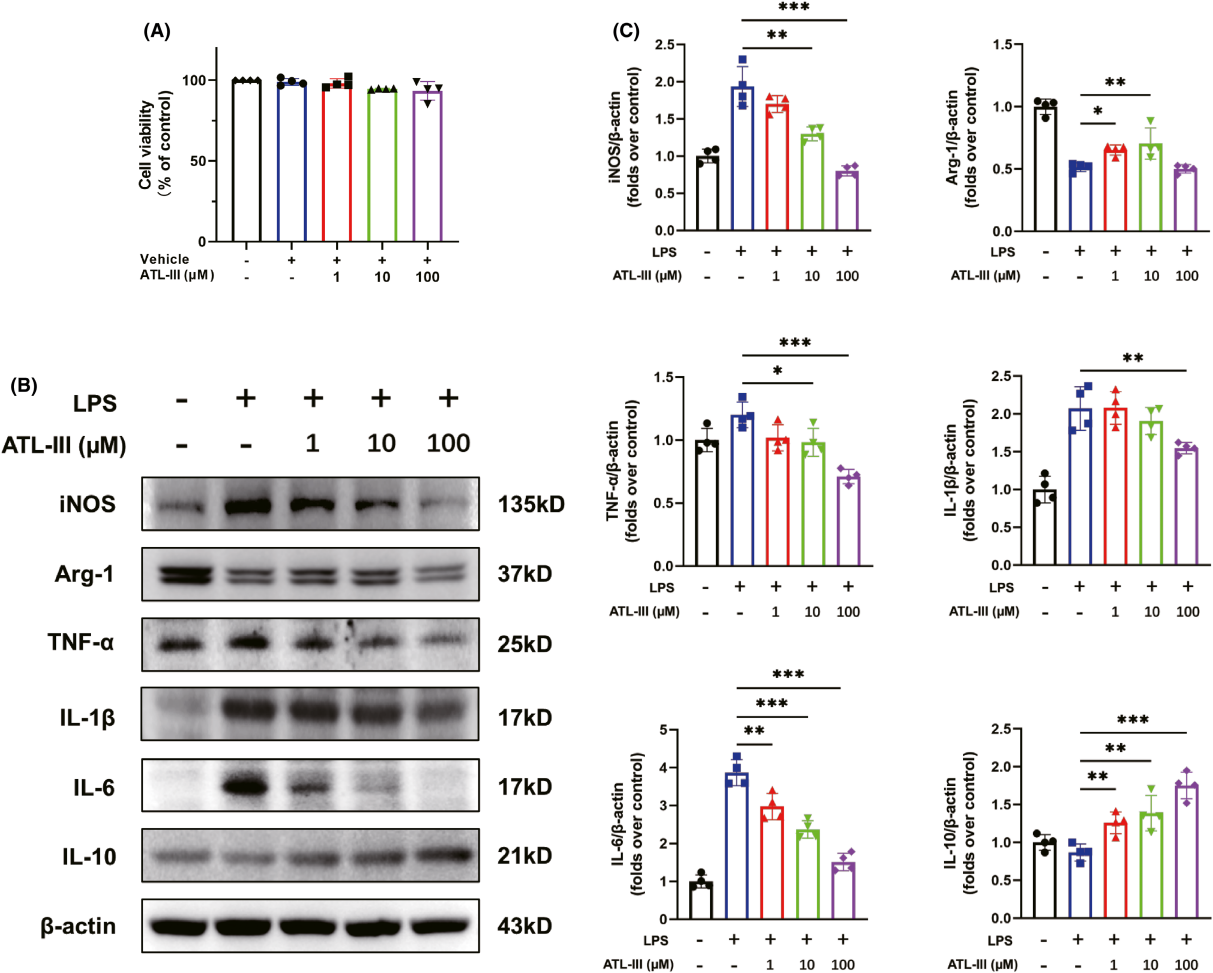
ATL‐III regulates the M1/M2 phenotype and the expression of corresponding inflammatory factors after LPS stimulation in microglia. (A) The viability of BV2 cells, as measured by the CCK‐8 assay. (B) Western blot analysis of iNOS, Arg‐1, inflammatory mediator (TNF‐α, IL‐1β and IL‐6), IL‐10 and β‐actin expression. (C) Quantitative analysis of the protein expression levels in microglia. The data are presented as the mean ± SD (*n* = 4). *p < 0.05, **p < 0.01, ***p < 0.001 compared with the control group

To explore whether ATL‐III directly regulates the M1/M2 phenotype and the expression of corresponding inflammatory factors in microglia, BV2 microglia were stimulated with LPS to confirm the data obtained in vivo. ATL‐III at doses of 1–100 μM did not obviously decrease the viability of microglia, as determined by the CCK‐8 assay (all *p* > 0.05; Figure 6A). The Western blot results showed that the level of iNOS was decreased in ATL‐III‐treated microglia after LPS stimulation, whereas the level of Arg‐1 was increased in response to ATL‐III administration (*p* < 0.05; Figure 6B,C). Moreover, ATL‐III inhibited the LPS‐induced upregulation of TNF‐α, IL‐1β, and IL‐6 expression and the downregulation of IL‐10 expression in microglia in a dose‐dependent manner (*p* < 0.05; Figure 6B,C). These data suggested that ATL‐III was not toxic to microglia, and observed trends in M1/M2 polarization and the levels of inflammatory factors were consistent with those observed in in vivo. Thus, ATL‐III could regulate the M1/M2 phenotype and the expression of corresponding inflammatory factors in microglia.

### 3.7 *Effects of ATL*‐*III on signaling pathways related to the polarization of microglia*

**FIGURE 7:**
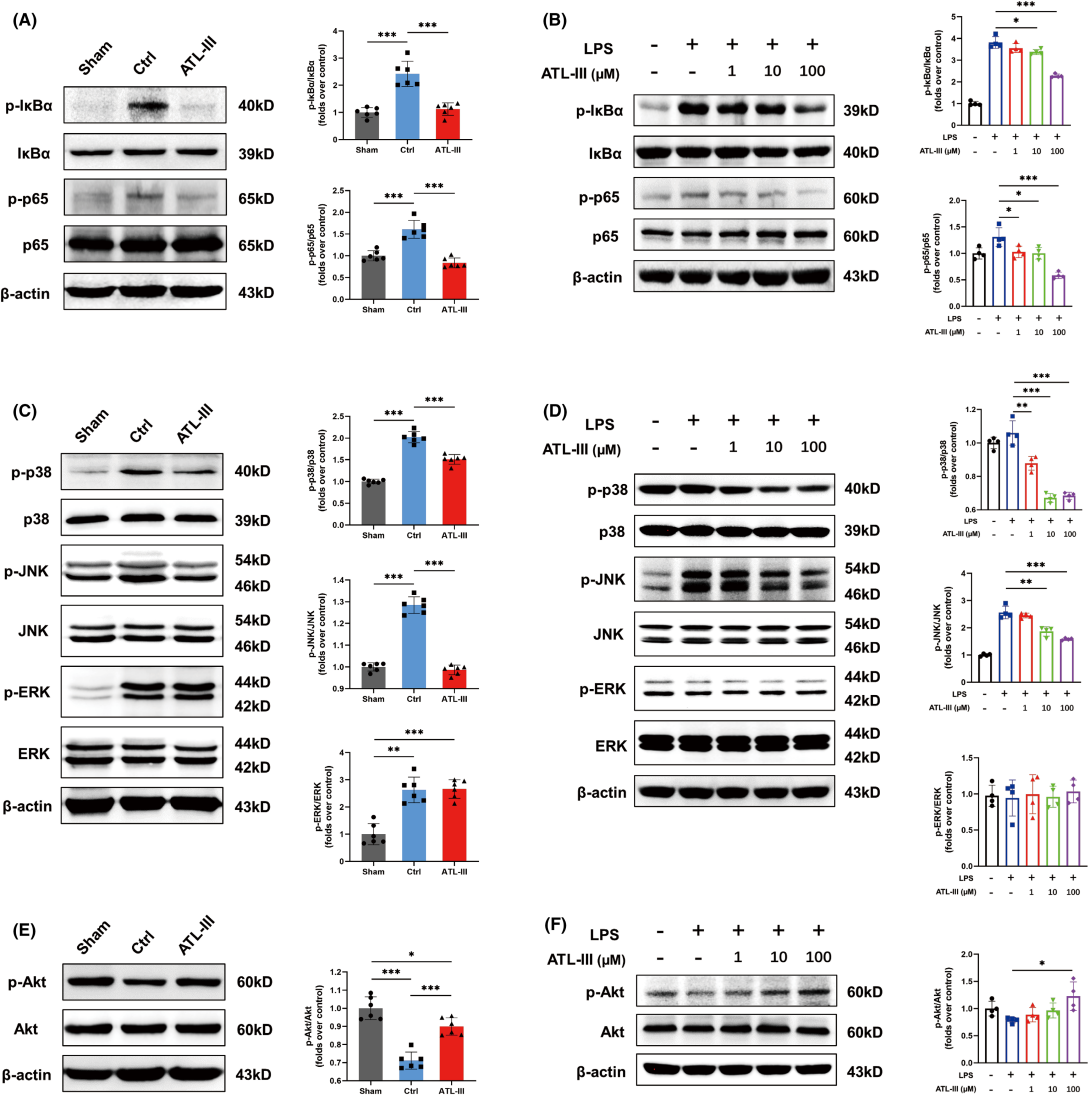
Effects of ATL‐III on the activation of the NF‐κB, MAPK, and Akt pathways. (A) Western blot analysis of the levels of p‐IκBα and p‐p65 in spinal cord tissues. (B) Western blot analysis of the levels of p‐IκBα and p‐p65 in microglia. (C) Western blot analysis of the levels of p‐p38, p‐JNK, and p‐ERK in spinal cord tissues. (D) Western blot analysis of the levels of p‐p38, p‐JNK, and p‐ERK in microglia. (E) Western blot analysis of the levels of p‐Akt in spinal cord tissues. (F) Western blot analysis of the levels of p‐Akt in microglia. The data are presented as the mean ± SD (*n* = 4 or 6). *p < 0.05, **p < 0.01 compared with the control group

To elucidate the specific mechanisms by which ATL‐III regulates microglial M1/M2 polarization and exerts antineuroinflammatory effects, the activation of the NF‐κB signaling pathway was analyzed by Western blotting. The levels of phosphorylated IκBα and p65, which are involved in the NF‐κB signaling pathway, were significantly attenuated by ATL‐III administration both in vivo and in vitro (*p* < 0.05; Figure 7A,B). JNK, p38, and ERK1/2 are known to activate NF‐κB, which participates in the expression of multiple proinflammatory factors.^24^, ^25^, ^26^ As shown in Figure 7(C,D), ATL‐III did not affect the expression of phosphorylated ERK1/2, which is involved in the MAPK signaling pathway, in vivo or in vitro, whereas the levels of phosphorylated JNK and p38 were suppressed by ATL‐III administration (*p* < 0.01). These data suggested that ATL‐III could partly inhibit the MAPK pathway and reduce the phosphorylation of molecules related to the NF‐κB pathway. The level of phosphorylated Akt as obviously increased after ATL‐III treatment (*p* < 0.001; Figure 7E). Likewise, we found that ATL‐III activated Akt phosphorylation in microglia after LPS stimulation (*p* < 0.05; Figure 7F). Together, these results could explain how ATL‐III could enhance the transformation from the M1 phenotype to the M2 phenotype.

## 4 DISCUSSION

SCI is a serious disabling disease and inflammation is the most critical pathologic change that occurs during the secondary injury phase.^27^ Previous reports have proven that microglia/macrophages can be divided into two main subgroups, namely M1 (proinflammatory) microglia/macrophages and M2 (anti‐inflammatory) microglia/macrophages.^28^, ^29^ Therefore, inflammation may be ameliorated by promoting the transformation of M1 into M2.^30^ However, most anti‐inflammatory drugs such as glucocorticoids and biological agents are not suitable for long‐term use because of their side effects or drug resistance.^31^

We have been greatly inspired to extract effective pharmaceutical compounds from natural plants to treat diseases. Some of these pharmaceutical compounds, such as artemisinin, which has been used for the treatment of malaria, have proven to be very successful.^32^ ATL‐III has multiple pharmacological properties and is virtually nontoxic even at high doses, and its high blood–brain barrier permeability makes it an ideal agent for treating neuroinflammation.^7^, ^33^ We hypothesize that ATL‐III exerts a neuroprotective effect by suppressing the activation and regulating the polarization of microglia/macrophages.

The severity of spinal cord tissue damage after SCI directly determines the extent of locomotor function recovery.^34^ The results showed that BBB scores and performance on the grid walk and footprint tests were obviously improved by ATL‐III administration. After confirming that motor function recovery was improved, we evaluated pathological changes at the tissue level. The results of HE, LFB, and Nissl staining showed that rats in the ATL‐III group had a significantly smaller lesion area, a larger LFB‐positive area and more residual motoneurons than the control group.

Inflammation is the key cause of secondary injury and affects motor function and histological outcome after SCI.^3^ According to our previous experiments, inflammation is most severe during the subacute phase (1–2 weeks) of SCI and M2 phenotype was transiently detected at high levels before 7 dpi.^35^ Thus, we investigated the effect of ATL‐III on the activation and polarization of microglia/macrophages at 7 days postinjury and found that the microglia of sham rats were inherent, with few but well‐proportioned M1 and M2 phenotypes. Following injury, a large number of microglia/macrophages were activated, with most of these activated cells being M1 phenotype, and ATL‐III treatment significantly decreased this activation. Our results demonstrate that ATL‐III could regulate the activation and polarization of microglia/macrophages in SCI rats. In general, upregulation of iNOS expression is considered a marker of activated M1 cells, while upregulation of Arg‐1 expression is considered a marker of activated M2 cells.^23^ The level of classic proinflammatory factors and anti‐inflammatory factors can be measured to determine the activity of M1 and M2 cells.^36^ After SCI, a large number of microglia are stimulated and change their morphology and function, mainly those related to the M1 phenotype. Similarly, LPS induces the polarization of microglia toward the M1 phenotype and increased the secretion of proinflammatory factors.^29^ These phenomena are consistent with what we observed in this study. Numerous studies have shown that inhibiting M1 polarization while inducing the transformation of microglia from the M1 phenotype to the M2 phenotype is more conducive to inhibiting neuroinflammation than simply inhibiting M1.^37^ Interestingly, ATL‐III decreased the level of iNOS, upregulated the expression of Arg‐1, and attenuated the inflammatory response both in vivo and in vitro. This could partly explain how ATL‐III inhibits neuroinflammation mediated by microglia by suppressing M1 polarization while promoting the transformation of cells from the M1 phenotype to the M2 phenotype.

We further identified the signaling events associated with ATL‐III‐mediated microglial polarization by determining the levels of NF‐κB, JNK MAPK, p38 MAPK, and Akt. In our research, ATL‐III treatment reduced the phosphorylation of IκBα and p65, which are involved in the NF‐κB signaling pathway, in SCI rats and in microglia stimulated with LPS. This finding is consistent with a previous study on the effect of ATL‐III in a human mast cell inflammation model.^38^ MAPK signaling also participates in activating the transcription factor NF‐κB.^25^, ^26^ Different studies have found that the activation of p38 and JNK contributes to the production of proinflammatory factors in activated microglia.^39^ In addition, p38 participates in the activation of M1 microglia by galectin‐1 and hierarchically inhibits downstream proinflammatory mediators, such as iNOS and TNF‐α.^40^, ^41^ In our study, the phosphorylation of JNK and p38 in MAPK signaling was significantly activated in spinal cord tissue and microglia after SCI or LPS stimulation, while ATL‐III treatment significantly inhibited the phosphorylation of JNK and p38. However, ATL‐III did not affect ERK1/2 phosphorylation in vivo or in vitro. Therefore, we speculate that the protective effect of ATL‐III against neuroinflammation after SCI is achieved at least in part by regulation of JNK MAPK and p38 MAPK. A growing number of reports have shown that the activation of the PI3K/Akt pathway can promote the polarization of microglia.^42^, ^43^ Therefore, we attempted to elucidate the role of ATL‐III in the phosphorylation of Akt in spinal cord tissue and microglia. We found that ATL‐III elevated the phosphorylation of Akt. These phenomena may explain how ATL‐III promotes the polarization of microglia, thereby ameliorating neuroinflammation after SCI and ultimately contributing to histological and functional recovery.

However, our study has a few limitations. We used female rats in our study. This is because female rats are more likely than male rats to artificially urinate due to anatomical differences in the urinary system. Therefore, the use of female rats reduces infection and mortality, while also reducing the impact of non‐injury factors on our experiments. It is undeniable that the sexual comparisons study may make our research more adequate. Our results show that ATL‐III alleviates secondary injury after SCI by regulating the polarization of microglia; however, ATL‐III may also ameliorate SCI through other means. The changes in NF‐κB, MAPK, and Akt signaling may partly explain the potential mechanism underlying the neuroprotection and the anti‐inflammatory effects of ATL‐III, but we may have overlooked other signaling pathways. In addition, the interactions between microglia/macrophages and other glial cells, such as oligodendrocytes that make up the myelin sheath, have not been exhaustively studied. Recent study has shown that microglia/macrophages could also clear myelin debris by phagocytosis, which creates a conducive environment for the regeneration of myelin.^44^ It seems likely that ATL‐III has multiple biological functions worth exploring in the future.

In conclusion, this study reveals a novel function for ATL‐III in the regulation of microglial polarization both in vivo and in vitro and provides initial evidence that ATL‐III has potential therapeutic benefits in promoting histological and functional repair in rats after SCI. This effect may be partly mediated via inhibiting of neuroinflammation induced by secondary injury through the NF‐κB, JNK MAPK, p38 MAPK, and Akt pathways. Given the established safety of ATL‐III, its protective effect in an animal model of SCI suggests that this compound may have clinical potential for human SCI.

## CONFLICT OF INTEREST

The authors declare no competing financial interests.

## AUTHOR CONTRIBUTIONS

JGH and HZL designed the experiments and edited the manuscript. MTX, WJS, and XS conducted the experiments and wrote the manuscript. YJS, ZJG, RW, LS, and RW conducted the data analysis and designed the figures. All authors read and approved the final manuscript.

## Supporting information

FigureS1
