# Supplementary material for: Atractylenolide III ameliorates spinal cord injury in rats by modulating microglial/macrophage polarization
Source: CNS Neurosci Ther. 2022 Apr 10;28(7):1059–71. doi: 10.1111/cns.13839 (PMC9160450; doi:10.1111/cns.13839)

Full unedited blot for Figure 5A

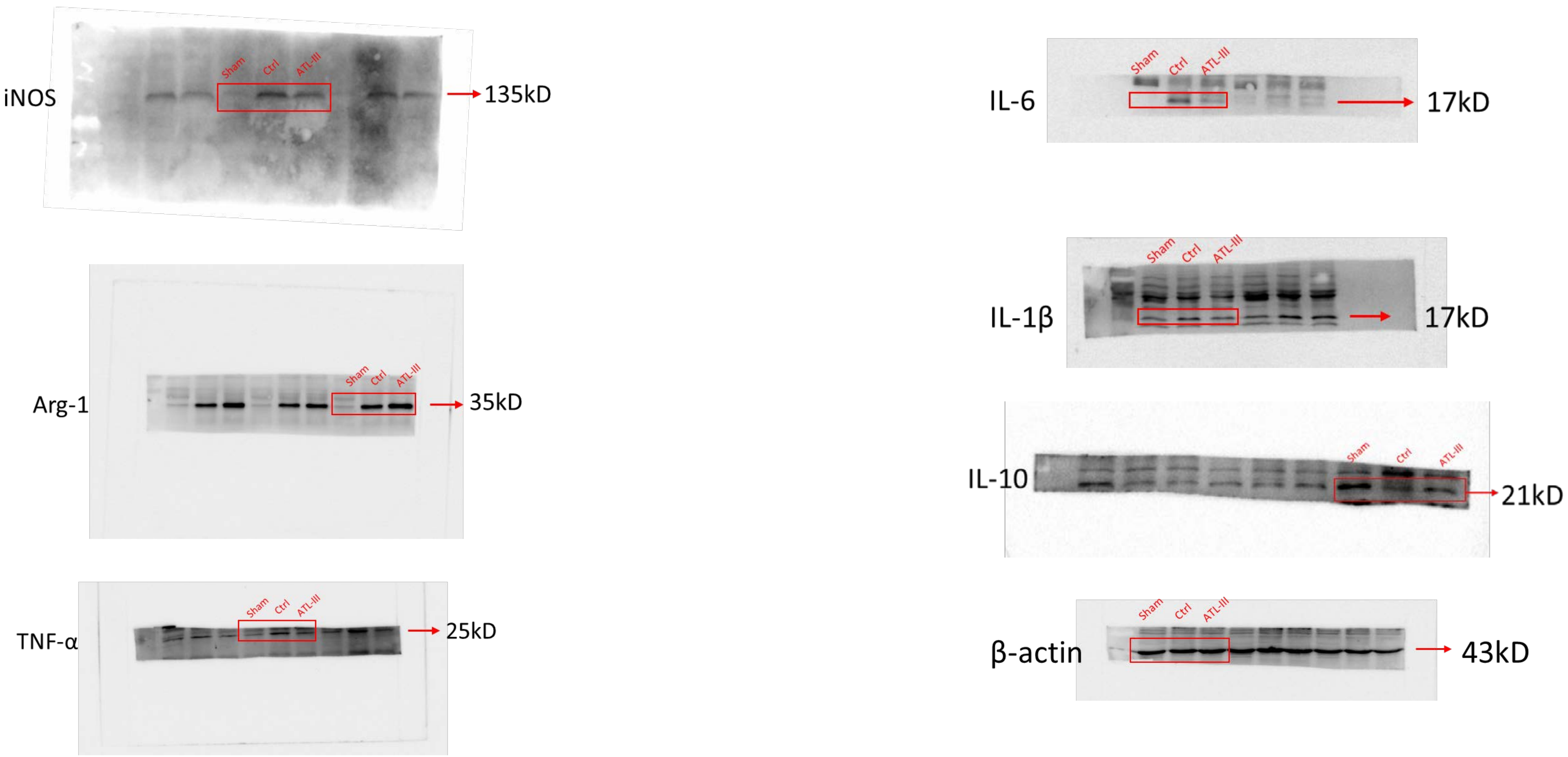

Full unedited blot for Figure 6B

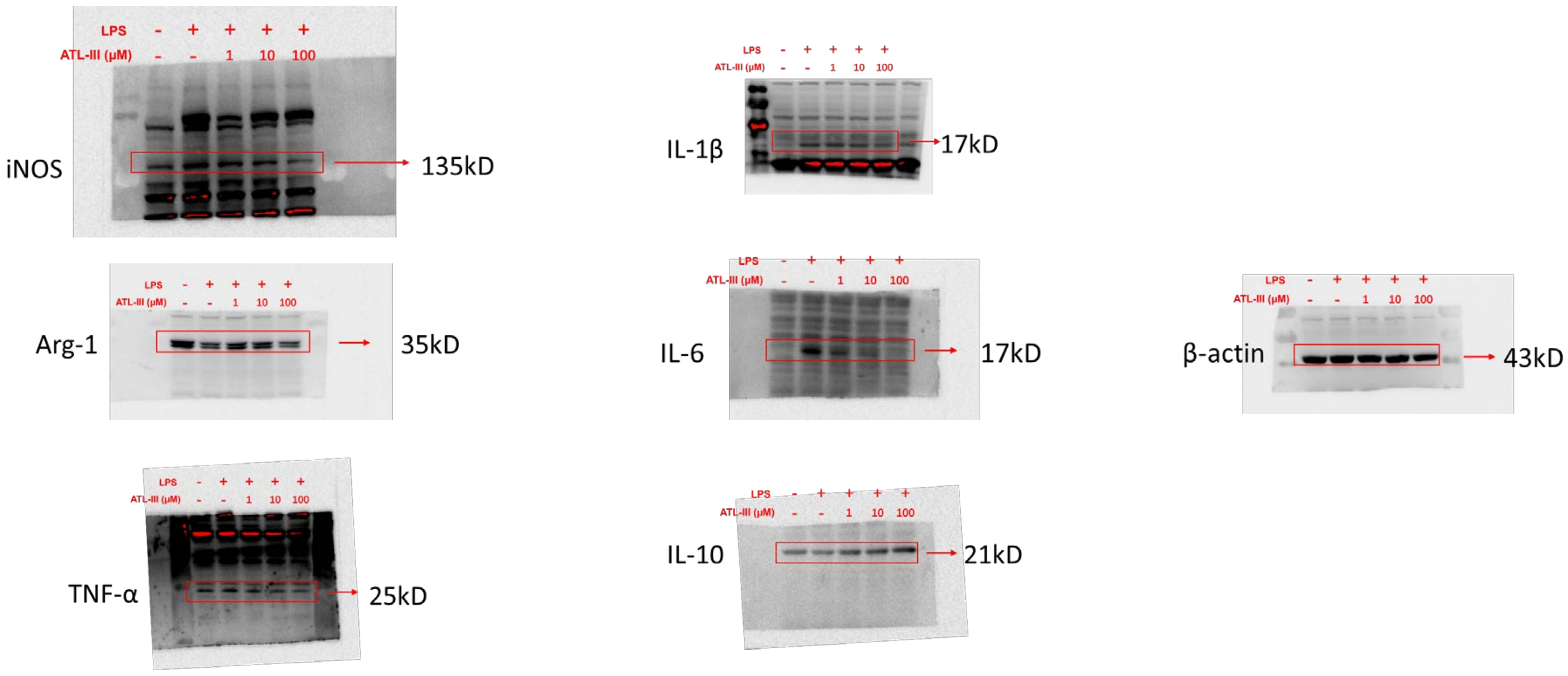

Full unedited blot for Figure 7A

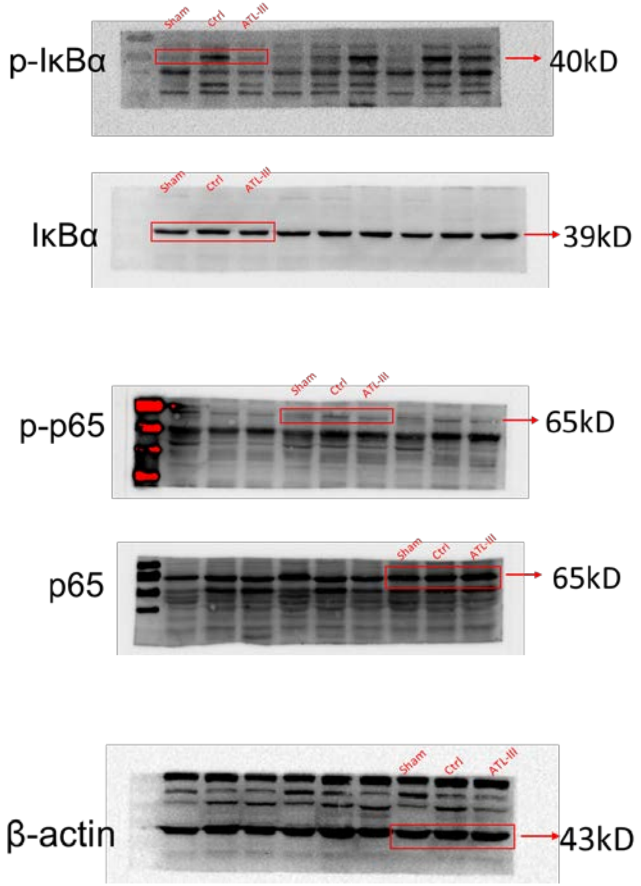

Full unedited blot for Figure 7B

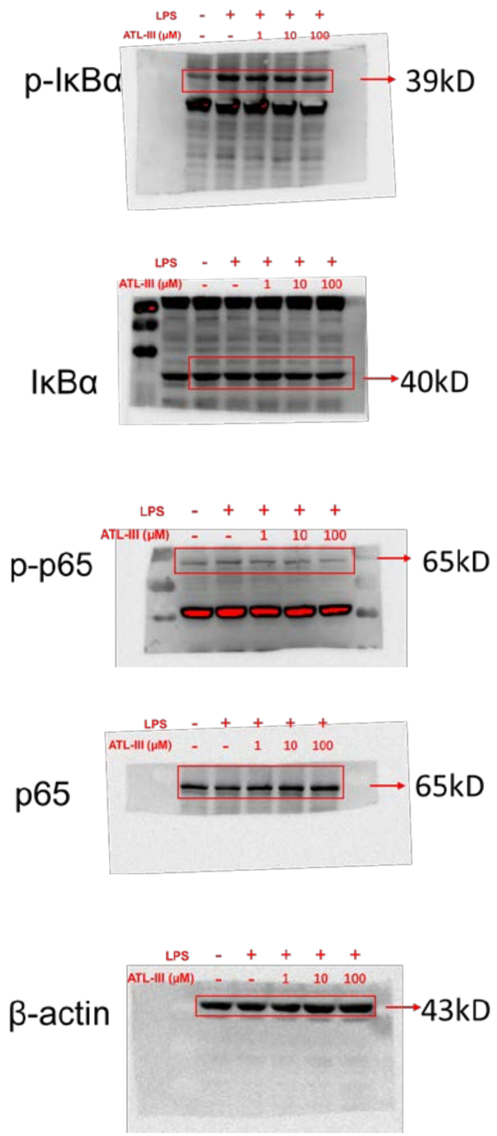

Full unedited blot for Figure 7C

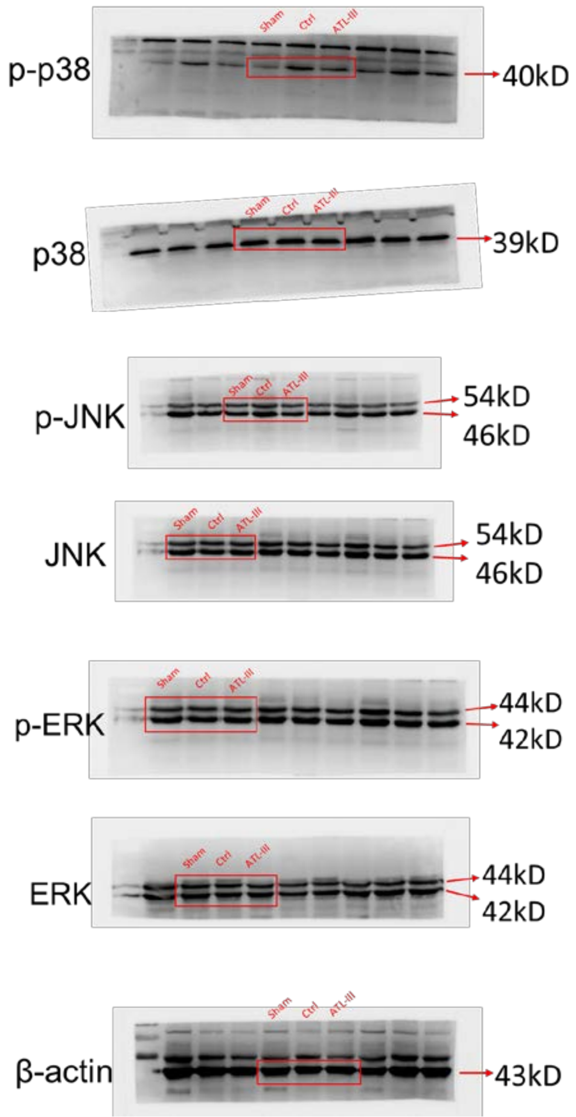

Full unedited blot for Figure 7D

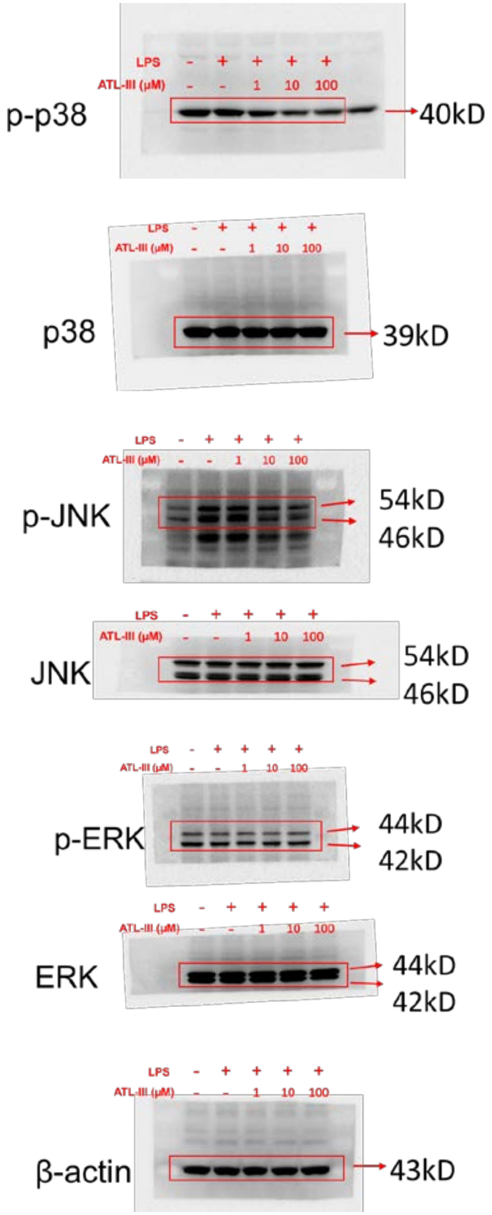

Full unedited blot for Figure 7E

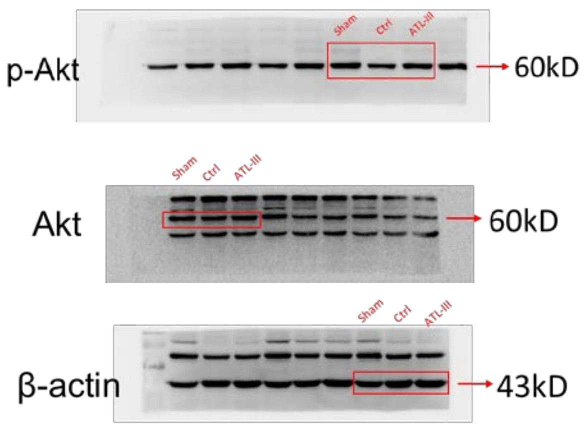

Full unedited blot for Figure 7F

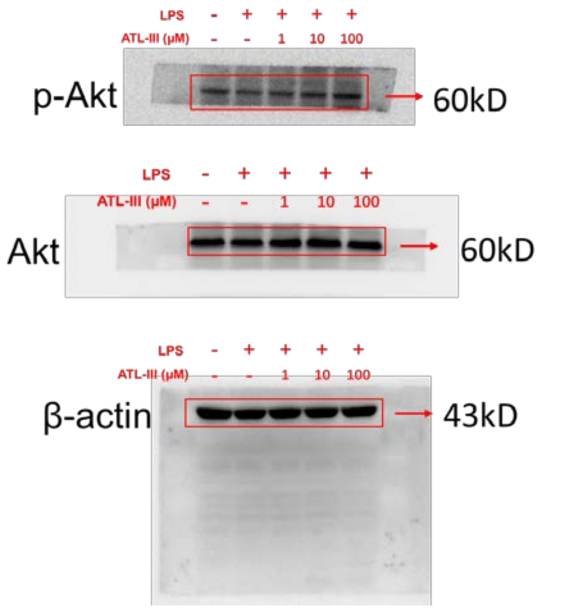

Supplemental Figure. S1

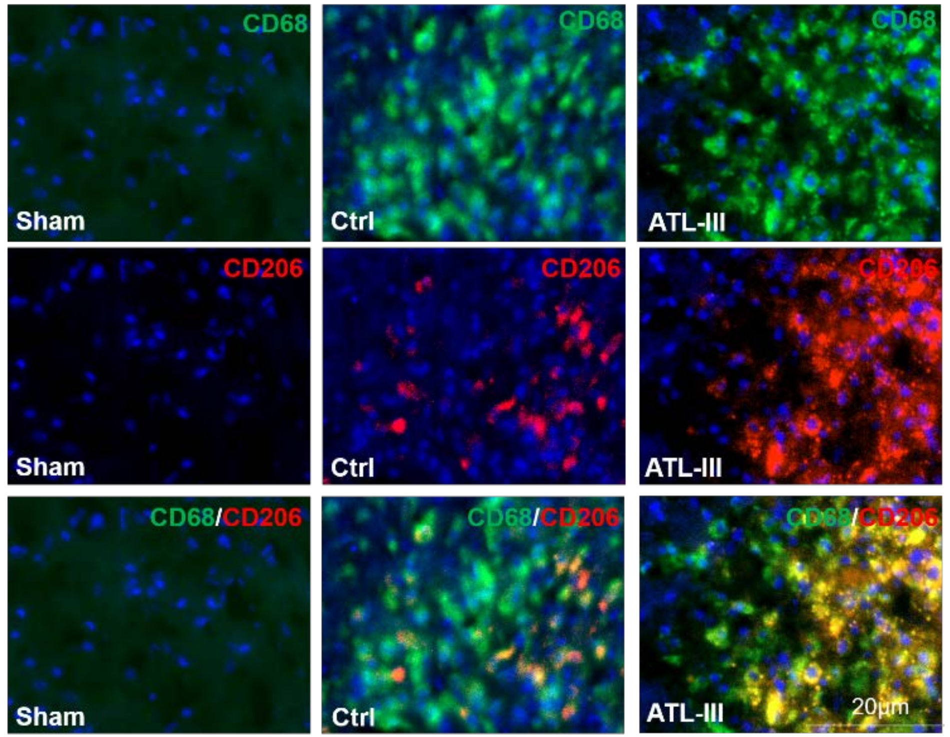

Supplemental Figure. S2

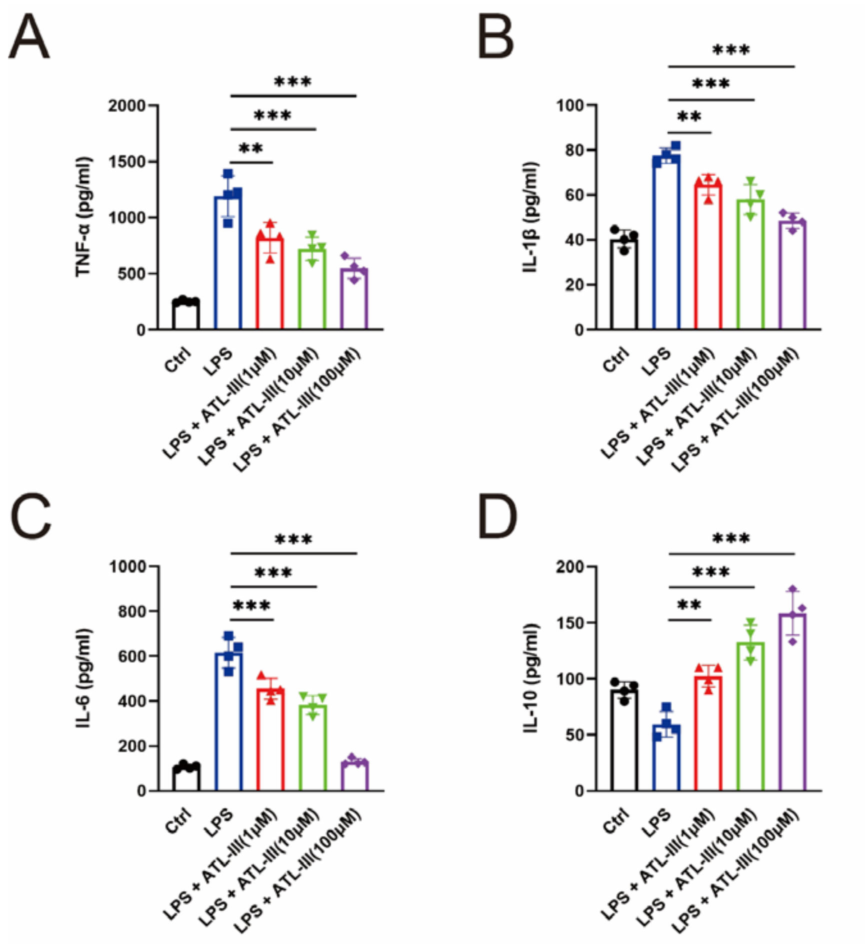

Supplement: Supplementary file 1 — FigureS1 [file CNS-28-1059-s001.pdf]
